# The Host-Protective Effect of Arabinosylated Lipoarabinomannan against *Leishmania donovani* Infection Is Associated with Restoration of IFN-γ Responsiveness

**DOI:** 10.1371/journal.pone.0117247

**Published:** 2015-02-06

**Authors:** Bidisha Paul Chowdhury, Syamdas Bandyopadhyay, Shibali Das, Saikat Majumder, Mukesh Kumar Jha, Suchandra Bhattacharyya Majumdar, Bhaskar Saha, Subrata Majumdar

**Affiliations:** 1 Division of Molecular Medicine, Bose Institute, Kolkata, India; 2 Laboratory-V, National Centre for Cell Science (NCCS), Pune, Maharashtra, India; University at Buffalo, UNITED STATES

## Abstract

Visceral leishmaniasis (VL), which is endemic as a major infectious disease in the tropical and subtropical countries, is caused by a protozoan parasite *Leishmania donovani*. At present, restricted treatment options and lack of vaccines intensify the problem of controlling VL. Therefore, finding a novel immunoprophylactic or therapeutic principle is a pressing need. Here, we report that arabinosylated lipoarabinomannan (Ara-LAM), a TLR2-ligand isolated from *Mycobacterium smegmatis*, exhibits a strong immunomodulatory property that conferred protection against *L. donovani* infection. Although, Ara-LAM modulates TLR2 and MAPK signaling, it is not known whether Ara-LAM involves IFN-γ signaling for effective parasite clearance. Because, it is reported that IFN-γ signaling, a principle mediator of NO generation and macrophage and Tcell activation, is hampered during leishmanial pathogenesis. Ara-LAM increases IFN-γ receptor expression and potentiates IFN-γ receptor signaling through JAK-STAT pathway. Moreover, Ara-LAM reciprocally modulates IRF4 and IRF8 expression and reinstates anti-leishmanial Th1 response that eventuates in significantly reduced parasite load in spleen and liver of *L. donovani*-infected BALB/c mice. IFN-γRα silencing resulted in the suppression of these host-protective mechanisms affected by Ara-LAM. Thus, Ara-LAM-mediated restoration of IFN-γ responsiveness is a novel immuno-modulatory principle for protection against *L. donovani* susceptible host.

## Introduction

Visceral leishmaniasis (VL), caused by a protozoan parasite *Leishmania donovani*, is one of the most widespread infectious diseases affecting many countries [1]. It is re-emerging as a major disease due to lack of effective chemotherapy and vaccine [2] and due to co-infection with HIV [3]. The HIV-*Leishmania* co-infection is virtually untreatable [4]. In addition, emergence of drug-resistant parasites [5] compels development of an immunotherapeutic principle. As *Leishmania* expressed lipophosphoglycan (LPG) interacts with the macrophage expressed TLR2 [6, 7], which subsequently regulates the expression and function of the leishmanial DNA recognizing TLR9 [8, 9], TLR2 is targeted for developing an anti-leishmanial immunotherapy.

We characterized a novel TLR2 ligand, arabinosylated-lipoarabinomannan (Ara-LAM)—a *Mycobacterium smegmatis* cell-wall glycolipid [10], for effective anti-tubercular immuno-prophylaxis [11]. Ara-LAM induces TLR2 expression and MyD88-dependent TLR2 signaling in *L*. *donovani*-infected macrophages [12]. Ara-LAM reciprocally regulates mitogen-activated protein kinase (MAPK) activation and pro-inflammatory cytokine production in a TLR2-dependent manner [13]. Among the pro-inflammatory cytokines, IFN-γ activates macrophages through JAK1/JAK2-STAT1 mediated signaling and induces nitric oxide synthase-dependent killing of amastigotes, the intracellular form of the parasite [14–16]. IFN-γ regulates several signaling molecules including Interferon Regulatory Factors (IRFs) [17]. IRFs comprise a very important group of transcription factors that modulate host defence through different interferon signaling [17–19]. Among all IRFs, IRF4 and IRF8 have been reported to play counteracting roles in the immune system, despite structural semblances and direct involvement in IFN-γ signaling. For example, IRF8 alone or in co-ordination with IRF1 regulates different IFN-γ-induced genes, such as IL-12, iNOS, MHC-II, TNF-α and NADPH phagocyte oxidase [20–23], which are implicated in anti-leishmanial defence [24]. In contrast, IRF4 was reportedly associated with Th2 response in *L*. *major* infection [25, 26]. During active infection, *L*. *donovani-*infected macrophages do not respond to IFN-γ suggesting that IFN-γ responsiveness is impaired [27]. As Ara-LAM induces proinflammatory response in infected macrophages and IRF8 acts as a link between TLR and IFN-γ signaling [28], it is quite possible that TLR2 ligand Ara-LAM modulates the IFN-γ induced signaling which in turn facilitates the anti-leishmanial effect of Ara-LAM. Thus, we investigated whether Ara-LAM, a TLR2 ligand, would restore IFN-γ responsiveness in *L*. *donovani*-infected macrophages and eliminate the parasite more efficiently.

Herein, we demonstrate that Ara-LAM pre-treatment transcriptionally activated the IFN-γ receptor α-chain (IFN-γRα) expression in *L*. *donovani-*infected macrophages resulting in increased expression of IFN-γRα on the cell surface and higher phosphorylation of JAK1, JAK2 and STAT1. This effect of Ara-LAM was found to be more pronounced than a known TLR2 ligand Peptidoglycan (PGN). The restored IFN-γ signaling, in turn, influenced IL-12 and IL-10 productions by reciprocally regulating the expressions of IRF8 and IRF4, followed by improved MHC-II expression on parasitized macrophages and robust T-cell proliferation. These effects of Ara-LAM conferred significant protection against *L*. *donovani* infection in susceptible BALB/c mice.

## Materials and Methods

### Ethics Statement

In this study, female and male BALB/c mice (18–20 gm) were used. The Animals were acclimatized for 15 days in polypropylene cages in the Animal House facility, with standard food and water *ad libitum*. The animal experiments were conducted in accordance with the OECD guidelines, accepted by the Committee for the Purpose of Control and Supervision on Experiments on Animals (CPCSEA), Thiruvanmiyur, Chennai, India, and as per the approval of the Institutional Animal Ethical Committee (Bose Institute, Kolkata, Registration Number: 95/99/CPCSEA). When required, the surgical procedures performed under Ketamine hydrochloride (100 mg/kg i.m.) anesthesia, and all efforts were made to minimize the suffering of the animals.

### Animals, macrophages and parasites

6–8 weeks old BALB/c mice (both sex) were purchased from the National Centre for Laboratory Animal Sciences, India. *L*. *donovani* (strain MHOM/IN/1983/AG83) were maintained in Medium 199 (Sigma) with 10% fetal calf serum (Gibco, Grand Island, NY) and passage through BALB/c mice to maintain the virulence. Stationary-phase promastigotes obtained by suitable transformation were used for infecting BALB/c mice and macrophages. Peritoneal macrophages from thioglycolated BALB/c mice were cultured for 48 h, as described elsewhere [29]. Adherent macrophages were infected with *Leishmania* promastigotes (stationary phase) at a ratio of 1:10. Experiments were performed according to the animal use protocols approved by the institutional animal ethics committee.

### Isolation and purification of Ara-LAM

Ara-LAM was isolated as described elsewhere [29]. The noncytotoxic dose of Ara-LAM was 3μg/ml [10].

### Preparation of TLR2-shRNA and IFN-γRα-shRNA

For in vivo gene silencing, TLR2-specific and IFN-γRα specific short hairpin oligos (shRNA~50 bases) were synthesized with a nine base loop sequences in the middle and a terminator sequence (five to six Ts) at the 3’-end and inserted in the multiple cloning site of pSilencer 1.0 U6 (mouse) plasmid vector having mouse U6 promoter (Ambion Inc., Grand Island, NY). Scrambled shRNA was used as Control shRNA.

### Preparation of cell lysate and immunoblot analysis

Cell lysates were prepared as described elsewhere [30]. Equal amounts of protein (50 μg) were subjected to 10% sodium dodecyl sulfate poly acrylamide gel electrophoresis, and immunoblotting was performed as described elsewhere [30].

### Flow cytometry

Macrophages were stained with phycoerythrin (PE)-labeled anti-IFN-γRα antibody and fluorescein isothiocyanate (FITC)–labeled anti-MHC-II antibodies (Santa Cruz Biotech). For intracellular cytokine staining, purified CD4^+^ T cells from soluble leishmanial antigen (SLA) stimulated and Brefeldin A (10 mg/ml) treated splenocytes and hepatocytes were permeabilized (0.1% saponin) and stained with anti-mouse IFN-γ-FITC and anti-mouse IL-10-PE antibodies (Santa Cruz Biotech, Santa Cruz, CA). Cells were analyzed using a FACS Verse flow cytometer (Becton Dickinson, San Diego, CA).

### Isolation of RNA and reverse transcriptase polymerase chain reaction

Total RNA extracted from macrophages (TRI reagent; Sigma) was reverse transcribed using Revert Aid M-MuLV reverse transcriptase (Fermentas) and semi-quantitative polymerase chain reaction (PCR) was performed using Perkin Elmer Gen Amp PCR system 2400. Glyceraldehyde-3-phosphate dehydrogenase (GAPDH) or β-actin were used as reference. Sequences of the PCR primers are listed in Table 1. The reaction conditions consisted of an initial activation step (5 min at 95°C) and cycling step (denaturation for 30 s at 94°C, annealing for 30 s at 58°C, and extension for 1 min at 72°C for 35 cycles). PCR amplified product was subsequently size fractioned on 2% agarose gel, stained with ethidium bromide and visualized under UV-light.

**Table 1:** Sequences of the PCR primers.

Gene	Sequences of primers
IL-12p40	Forward 5’-ATTGACTGGCGTTGGAAGCA-3’
	Reverse 5’-TGCGCTGGATTCGAACAAAGA-3’
IFN-γ	Forward 5’-AGCTCTTCCTCATGGCTGTTTC-3’
	Reverse 5’-TGTTGCTGATGGCCTGATTGT-3’
IL-10	Forward 5’-ACTTGGGTTGCCAAGCCTTAT-3’
	Reverse 5’-ATCACTCTTCACCTGCTCCACT-3’
iNOS	Forward 5’-CCTGACTGAAGCACTTTGGGTG-3'
	Reverse 5’-TACAGGAAAGGCCCAAGCCAT-3'
IRF-1	Forward 5’- CAGAGGAAAGAGAGAAAGTCC-3’
	Reverse 5’- CACACGGTGACAGTGCTGG-3’
IRF-2	Forward 5’- CAGTTGAGTCATCTTTGGGGC-3’
	Reverse 5’- TGGTCATCACTCTCAGTGG-3’
IRF-3	Forward 5’- TACGTGAGGCATGTGCTGA-3’
	Reverse 5’- AGTGGGTGGCTGTTGGAAAT-3’
IRF-4	Forward 5’- GTGACTGTGCCCTGGCTTAT-3'
	Reverse 5’- TGGACATGATCTGGGCAACC-3'
IRF-5	Forward 5’- AATACCCCACCACCTTTTGA-3’
	Reverse 5’- TTGAGATCCGGGTTTGAGAT-3’
IRF-6	Forward 5’- GATGTACGATGGCACCAAGG-3’
	Reverse 5’- ACCGTTGATGTTCAGGAAGG-3’
IRF-7	Forward 5’- TGCAGAAGGTGGTGGGACA-3’
	Reverse 5’- TGCTATCCAGGGAAGACACA-3’
IRF-8	Forward 5’-AACTGTGCTCTGGGCTCATC-3'
	Reverse 5’- CCTCCGGGAAGTGTCCCTTA-3'
IFN-γRα promoter	Forward 5’- GCTGCCAGTCTTCTAGTGGGA-3’
	Reverse 5’- TCCTTGAGAAGTGCTGCTGGG-3’
IL-12p40 promoter	Forward 5’-AAATTCCCCCAGAATGTTTTGACAC-3’
	Reverse 5’-CGAGCTGCCTGGTCTGATGT-3’
IL-10 promoter	Forward 5’-GACCTGGGAGTGCGTGAATGG-3’
	Reverse 5’-AGCGCTAAAGAACTGGTCGGAATG-3’
GAPDH	Forward 5’-GTTGTCTCCTGCGACTTCAACA-3’
	Reverse 5’-TCTCTTGCTCAGTGTCCTTGCT-3’
β-actin	Forward 5’-GATGATATCGCCGCGCTCGT-3’
	Reverse 5’-GTAGATGGGCACAGTGTGGGTG-3’

### Preparation of cytosolic and nuclear extracts

Cells were collected after incubation for indicated periods. The cytosolic and nuclear fractions were seperated as described elsewhere [12].

### Chromatin Immuno-precipitation (ChIP) assay

CHIP assays were conducted using the CHIP Assay kit following the manufacturers Protocol (Millipore, Billerica, MA) as described elsewhere [13]. With the extracted DNA, PCR was conducted using promoter specific primers: IFN-γRα promoter (NF-κB binding site), IL-10 promoter (IRF4 binding site) and IL-12p40 promoter (IRF8 binding site) (Table 1). PCR amplified product was resolved on 2% agarose gel, stained with ethidium bromide and visualized under UV-light.

### In vitro parasite burden count

For studying the activity of Ara-LAM against the uptake and intracellular multiplication of *L*. *donovani* parasite, peritoneal macrophages cultured on glass cover slips were treated and infected as per protocol; macrophages were then fixed and stained with Giemsa as described elsewhere [13] for calculation of the number of intracellular amastigotes.

### In vivo experiments

BALB/c mice were divided in seven groups according to treatment regime: (1) control mice (administered phosphate-buffered saline); (2) *L*. *donovani*–infected mice; (3) Ara-LAM–treated mice (injected with 30 μg of Ara-LAM intraperitonially); (4)Ara-LAM–treated infected mice (injected with 1x10^7^ parasites/mice, through tail vein after 2 days of Ara-LAM treatment); (5) TLR2-shRNA administered Ara-LAM–treated infected mice; (6) IFN-γRα-shRNA administered Ara-LAM–treated infected mice; and (7) Control shRNA administered Ara-LAM–treated infected mice. TLR2-shRNA, IFN-γRα-shRNA or control shRNA (100μg/mice) were administered through tail vein. After 2 days, mice were subjected to Ara-LAM (30μg/mice, i.p.) treatment for another 2 days followed by *L*. *donovani* infection (1x10^7^ parasites/mice, i.v). Mice were sacrificed 28 days after infection; the splenic and hepatic parasite loads (expressed in Leishman-Donovan units) were enumerated under a microscope [12]. Isolated splenocytes were cultured in RPMI 1640 medium plus 10% (FCS) for cytokine profiling, gene expression study and T-cell proliferation assay.

### Cytokine enzyme-linked immunosorbent assay

Culture supernatants were analyzed using a sandwich enzyme-linked immunosorbent assay kit (BD Biosciences), in accordance with the manufacturer’s instructions.

### Nitrite generation

Nitrite level in culture was measured using the Nitric Oxide Colorimetric Assay kit (Boehringer Mannheim Biochemicals), as described elsewhere [12].

### Proliferation assay

The T cell proliferation assay was performed as described elsewhere [12]. ^3^H-Thymidine uptake, as an index of proliferation, was measured using a liquid scintillation counter (Tri-Carb 2800TR; Perkin Elmer).

### CD4^+^ T cell purification

Splenic and hepatic CD4^+^ T-cells (purity ~95% as ascertained by FACS) from the indicated mice were isolated by positive selection using CD4^+^ IMag beads, according to the manufacturer’s instructions (BD Biosciences).

### Statistical analysis

In vitro assays were done in triplicate and a minimum of 4 mice were used per group for in vivo experiments. Data, including densitometry analysis, shown as means ± SD, are from 1 experiment performed at least 3 times. Student's *t*-test or one-way ANOVA was used to assess the significance of differences between the mean values for control and experimental groups. A difference with *P* <0.05 was considered significant, and a difference with *P* < 0.001 was considered highly significant.

## Results

### 1. Ara-LAM enhanced IFN-γ Rα expression in *L*. *donovani*-infected macrophages

**Fig 1:**
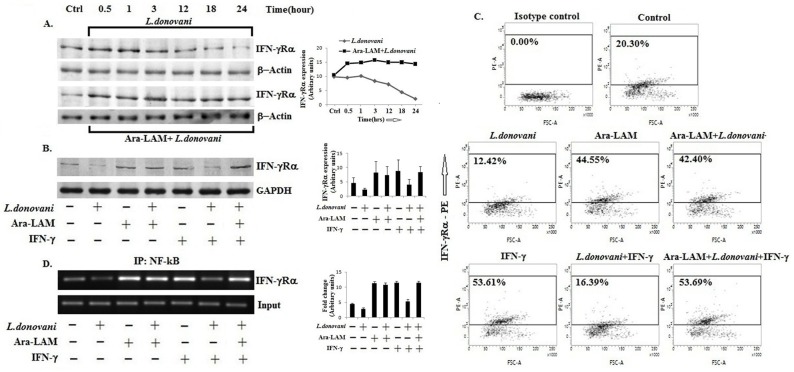
Ara-LAM enhanced IFN-γ Receptor alpha chain expression in parasitized macrophages. (A) BALB/c derived peritoneal macrophages (2x10^6^cells) were infected with *Leishmania donovani* (cell: parasite 1:10) for 30mins, 1hr, 3hrs, 12hrs, 18hrs and 24hrs. Other sets of macrophages were pre-treated with Ara-LAM for 3 hrs and followed by infection for the same time periods. Cell lysates were prepared followed by Western blot for IFN-γRα. Data are from 1 of 3 experiments conducted in the same way with similar results. IFN-γRα expression in Ara-LAM treated infected macrophages were significantly higher than the infected cells (p<0.05). (B) In a separate set of experiment, BALB/c derived peritoneal macrophages (2x10^6^cells) were pre-treated with Ara-LAM for 3 hr and followed by *Leishmania donovani* infection for 24 hr. Macrophages were then treated with rIFN-γ (20ng/ml) for 45min, followed by cell lysate preparation and Western blotting for IFN-γRα. The blots shown are representative of three experiments. (C) Ara-LAM pre-treated peritoneal macrophages (2x10^6^cells) were infected with *L*. *donovani* for 24 hrs followed by rIFN-γ (20ng/ml) stimulation for another 24 hrs, and staining with IFN-γRα-PE Ab and analyzed on a flow cytometer. Data are from one representative experiment that was repeated thrice. (D) Treated and untreated infected macrophages (2x10^6^cells) were subjected to immuno-precipitation using NF-κB (IP:NF-κB) specific Ab. Semi quantitative RT-PCR was performed for amplifying the putative NF-κB binding sites at the IFN-γRα promoter. Data are from one representative experiment, which was performed at least thrice.

In chronic leishmanial infections, host macrophages lose IFN-γ responsiveness due to impaired IFN-γ receptor expression and signalling [31–34]. We examined whether Ara-LAM pre-treatment would enhance IFN-γRα expression in BALB/c-derived thioglycolate-elicited *L*. *donovani*-infected macrophages. We observed that the IFN-γRα expression started decreasing 3 hours after the infection (Fig. 1A) and Ara-LAM prevented the reduction in IFN-γRα expression in *L*. *donovani*-infected macrophages (Fig. 1A, 1B, 1C; p< 0.05) possibly by enhancing NF-κB binding to IFN-γRα promoter (Fig. 1D). The Ara-LAM enhanced IFN-γRα expression was further potentiated by rIFN-γ stimulation (Fig. 1B, 1C, 1D), suggesting that Ara-LAM primes the macrophages to respond to IFN-γ and enhance IFN-γ responsiveness of the infected macrophages. These findings suggest that the Ara-LAM enhanced IFN-γRα expression may restore IFN-γ responsiveness of the parasitized macrophages.

### 2. Ara-LAM increased IFN-γ responsiveness in *L*. *donovani*-infected macrophages

**Fig 2:**
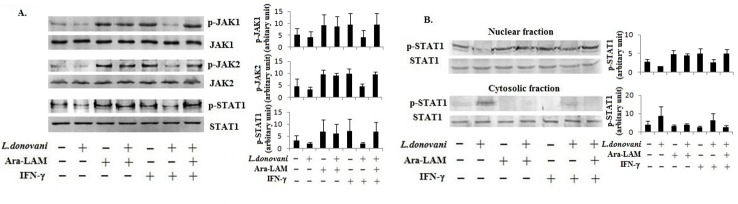
Moderation of IFN-γ induced JAK-STAT signaling by Ara-LAM in *Leishmania* infected macrophages. (A) BALB/c derived peritoneal macrophages were pre-treated with Ara-LAM for 3 hrs, followed by *L*. *donovani* infection for 24hrs. Macrophages were treated with rIFN-γ (20ng/ml) for 45min, followed by cell lysate preparation and Western blot for JAK1, p-JAK1, JAK2, p-JAK2, STAT-1 and p-STAT-1. The blots shown are representative of triplicate experiments. (B) Both the nuclear extracts and the cytosolic extracts of differently treated peritoneal macrophages were prepared followed by Western blot to analyze the nuclear translocation of p-STAT-1 in *L*. *donovani* infected macrophages. Blots shown here are from one of three representative experiments.

IFN-γ responsiveness depends not only on IFN-γR expression but also on the effective IFN-γ signaling through JAK1/JAK2-STAT1 pathway [32, 33]. Therefore, we assessed IFN-γ-induced JAK/STAT phosphorylation in Ara-LAM-treated parasitized macrophages. We observed that Ara-LAM restored the rIFN-γ-induced JAK1, JAK2 and STAT1 phosphorylation, which had been suppressed by *L*. *donovani* infection (Fig. 2A). As a result, active STAT1 was increased in the nuclear fraction of the Ara-LAM pre-treated infected macrophages compared to the untreated, *L*. *donovani*-infected macrophages (Fig. 2B) indicating that Ara-LAM enhanced IFN-γ responsiveness in *L*. *donovani*-infected macrophages by restoring IFN-γ signaling through JAK-STAT pathway. Moreover, we observed that Ara-LAM showed more potent induction of IFN-γRα expression and its downstream signaling through JAK1/JAK2 and STAT1 activation in *L*. *donovani* infected macrophages, in comparision with the known TLR2 ligand Peptidoglycan (PGN) (S1 Fig.).

### 3. Ara-LAM differentially regulated Interferon Regulatory Factors in infected macrophages

**Fig 3:**
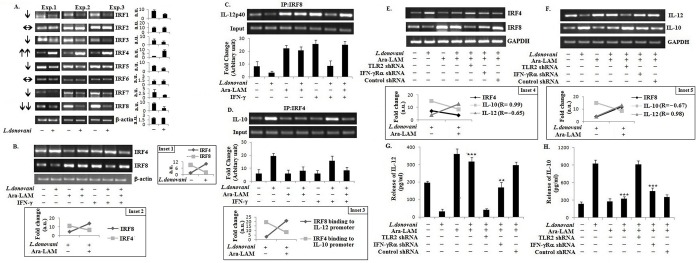
Ara-LAM reciprocally regulated IRF4 and IRF8 expression and corresponding immune response during *L*. *donovani* infection both in vitro and in vivo. (A) *L*. *donovani* infected and uninfected control peritoneal macrophages were collected in Trizol for RNA extraction and semi-quantitative RT-PCR was performed. Data represent means ± SD for three experiments. **Inset 1**: Comparison between IRF4 and IRF8 expression in *L*. *donovani* infected and uninfected control macrophages. (B) Peritoneal macrophages (2x10^6^cells) from BALB/c mice were cultured and subjected to Ara-LAM pre-treatment (3μg/ml) for 3hrs, followed by *L*. *donovani* challenge for 6hrs. Both rIFN-γ stimulated (20ng/ml, 45mins) and unstimulated cells were collected in Trizol for RNA extraction and semi-quantitative RT-PCR was performed. Data are from one representative experiment, which was performed at least thrice. **Inset 2:** Reciprocal expression of IRF4 and IRF8 by Ara-LAM in *L*. *donovani* infected macrophages. (C-D) BALB/c derived peritoneal macrophages (2x10^6^cells) were treated with Ara-LAM (3μg/ml) for 3hrs, followed by *L*. *donovani* challenge for 6hrs. After 45 min of rIFN-γ stimulation immuno-precipitations were conducted using IRF8 (IP:IRF8) and IRF4 (IP: IRF4) specific Abs. Semi quantitative RT-PCR was performed for amplifying the putative IRF8 binding sites of the IL-12p40 promoter and IRF4 binding sites of the IL-10 promoter. Data represent means ± SD for three sets of experiments. **Inset 3:** Ara-LAM mediated up-regulation of IRF8 binding to the IL-12 promoter and down-regulation of IRF4 binding to the IL-10 promoter in infected macrophages. (E-F) BALB/c mice were injected with respective shRNAs (for 2 days) followed by treatment with either phosphate-buffered saline (PBS) (control) or Ara-LAM (30 μg intraperitoneally) for 2 days, after which mice were infected. 28 days later, mice were sacrificed and splenocytes (2x10^6^) were collected in Trizol for mRNA extraction and semi-quantitative reverse transcriptase polymerase chain reaction (RT-PCR) analysis (see Methods). Data are from one of three representative experiments. **Inset 4:** Correlations (R-values) between IRF4 and IL-10 or IL-12 expression in Ara-LAM treated and untreated infected splenocytes. **Inset 5:** Correlations (R-values) between IRF8 and IL-10 or IL-12 expression in same set of mice. (G-H) A separate set of splenocytes (2x10^6^) were stimulated with soluble leishmanial antigen (SLA) at 5 μg/ml for 48 h. Release of Interleukin 12 (IL-12) p70 and Interleukin 10 (IL-10) culture supernatants were determined by enzyme-linked immunosorbent assay. Data represent means ± SD for 4 animals per group. ***P <.001 and **P < .01 for the comparison with infected mice.

As IFN-γ responsiveness also depends on Interferon Regulatory Factors (IRFs) [24, 35, 36], we examined the effects of Ara-LAM on the expression of these IRFs in *L*. *donovani*-infected macrophages. We observed that *L*. *donovani* differentially modulated the expression of eight IRFs in macrophages (Fig. 3A). Of these, IRF4 expression increased but IRF8 expression decreased during infection (Fig. 3A). Ara-LAM reversed the expression of IRF8, which is implicated in Th1 response [20–23], and IRF4, which facilitates Th2 response [37], in *L*. *donovani*-infected macrophages (Fig. 3B).

To further confirm the involvement of Ara-LAM in the regulation of IRF function (IRF4 and IRF8), we performed ChIP assay at IL-12p40 and IL-10 loci. We observed that Ara-LAM augmented the rIFN-γ induced IRF8 binding to the IL-12p40 promoter in parasitized macrophages (Fig. 3C). By contrast, Ara-LAM pre-treatment significantly reduced the binding of IRF4 to the IL-10 promoter in the *L*. *donovani*-infected macrophages (Fig. 3D).

Corroborating with these data, Ara-LAM induced IRF8 expression but suppressed IRF4 expression (Fig. 3E), accompanied by increased IL-12 but diminished IL-10 production, in *Leishmania*-infected BALB/c mice (Fig. 3F, 3G, 3H; R = 0.98 for correlation between IRF8 and IL-12, and R = 0.99 for correlation between IRF4 and IL-10). TLR2 shRNA abrogated IRF8 and IL-12 expression but elevated IRF4 and IL-10 expression in Ara-LAM treated BALB/c mice; these effects of Ara-LAM were significantly suppressed in IFNR silenced condition (Fig. 3E–3H). Thus, Ara-LAM reciprocally modulated IRF4 and IRF8 transcription factors that might mediate the anti-leishmanial effect of Ara-LAM.

### 4. Host-protective effects of Ara-LAM was abrogated by IFN-γRα or TLR2 silencing

**Fig 4:**
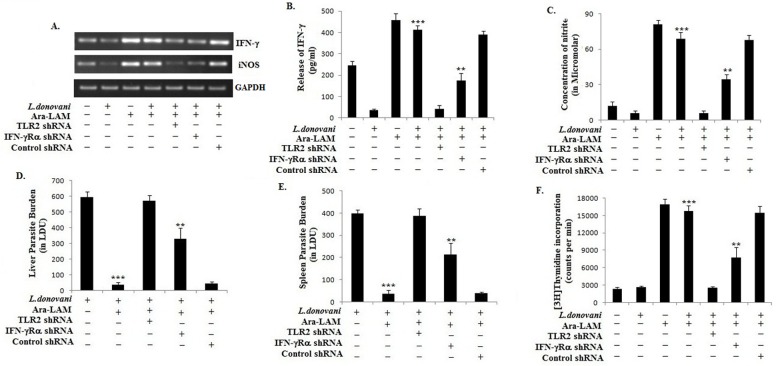
Effect of Ara-LAM and IFN-γ signaling against leishmanial pathogenesis in BALB/c mice. (A) Mice were injected with respective shRNAs (for 2 days) followed by treatment with either phosphate-buffered saline (PBS) (control) or Ara-LAM (30 μg intraperitoneally) for 2 days, after which mice were infected. After 28 days mice were sacrificed, splenocytes (2x10^6^) was collected in Trizol for mRNA extraction and semi-quantitative reverse transcriptase polymerase chain reaction (RT-PCR) analysis (see Methods). Data are from one of three representative experiment. (B-C) A separate set of splenocytes (2x10^5^cells/well) was stimulated with soluble leishmanial antigen (SLA) at 5 μg/ml for 48 h. Release of Interferon-γ (IFN-γ) and nitric oxide in culture supernatants were determined by enzyme-linked immunosorbent assay and the Nitric Oxide Colorimetric Assay kit, respectively. Data represent means ± SD for four animals per group. ***P <.001 and **P <. 01 for the comparison with infected mice. (D-E) Differently treated infected mice were sacrificed 28 days after infection. Levels of parasite burden in liver and spleen are expressed in Leishman-Donovan units (LDUs). Data represent means ± SD for 4 animals per group. ***P <.001 and **P < .01 for the comparison with infected mice. (F) Proliferative responses to soluble leishmanial antigen (SLA) (5 μg/ml) of splenocytes from above mentioned group of mice were examined by measuring [^3^H]thymidine incorporation. At 5 μg/ml SLA, optimal proliferation was obtained. Data represent means ± SD for 4 animals per group. ***P <.001 and **P < .01 for the comparison with infected mice.

As Ara-LAM, a TLR2 ligand, restored IFN-γ responsiveness in *L*. *donovani*-infected macrophages in vitro, we examined whether Ara-LAM protected *L*. *donovani*-infected BALB/c mice and if protected, whether the protection was dependent on TLR2 or IFN-γR. So, here we used two specific shRNAs for TLR2 and IFN-γRα respectively and their silencing efficacy was checked at both mRNA and protein level (S2 Fig.). We observed that silencing of TLR2 and IFN-γRα with TLR2-shRNA and IFN-γRα-shRNA, respectively, significantly reduced the Ara-LAM induced IFN-γ and nitric oxide production in *L*. *donovani*-infected BALB/c mice (Fig. 4A, 4B, 4C). The suppressed NO generation was correlated with the decreased iNOS expression in the spleen cells of these shRNA administered infected mice (Fig. 4A). The diminished IFN-γ and NO generation corroborated with the failure of Ara-LAM to reduce hepatic or splenic parasite burden in those IFN-γR, or TLR2, silenced BALB/c mice (Fig. 4D, 4E). For further confirmation, this anti-parasitic effect of Ara-LAM was compared with that of PGN and found Ara-LAM to be more potent in reducing parasite burden in *L*. *donovani* infected BALB/c mice (S3 Fig.).

Because the elevated production of Th1 cytokines is essential for the activation and proliferation of T cells for anti-leishmanial protection whereas impaired or deviated T cell response is required for *Leishmania* survival [38, 39], we investigated whether Ara-LAM treatment could restore the *Leishmania* impaired T-cell proliferation. We observed that Ara-LAM rectified the deficient T-cell proliferation in *L*. *donovani*-infected control shRNA treated but not in TLR2 silenced or IFN-γRα silenced mice (Fig. 4F). These results indicated that Ara-LAM restored T cell proliferation in *L*. *donovani*-infected mice in TLR2 and IFN-γRα dependent manner.

### 5. Ara-LAM reversed the T-helper subset expansion in *L*. *donovani*-infected mice

**Fig 5:**
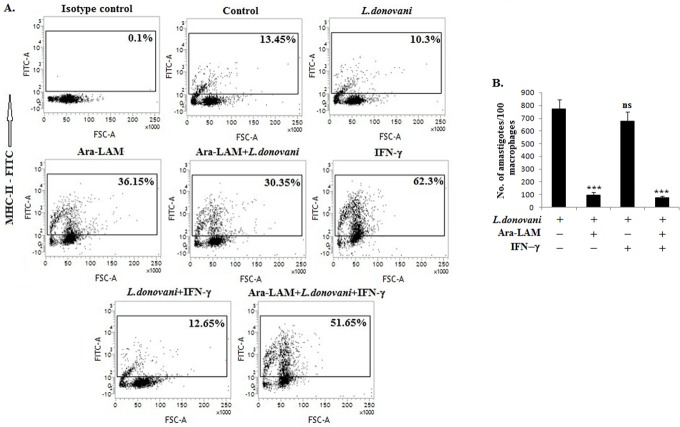
Ara-LAM induced the MHC-II expression in the membrane of *L*. *donovani* infected cells and helped in parasite killing. (A) Peritoneal macrophages (2x10^6^cells) isolated from BALB/c mice were pre-treated with Ara-LAM (3μg/ml) for 3hrs, followed by *Leishmania donovani* challenge for 24hrs. Both rIFN-γ (20ng/ml) stimulated (for 24hrs) and non-stimulated cells were then stained with anti-MHCII-FITC antibody and analyzed by Flow cytometry for MHC-II. Data are from one of three representative experiments. (B) In a similar set of experiment, the macrophages were cultured in cover slips, treated with Ara-LAM for 3 hrs followed by *Leishmania* infection and rIFN-γ stimulation (20ng/ml). After 24hrs of incubation intracellular parasite number were assessed as described in methods. Data represent means ± SD for three sets of experiments. ***P <.001 for the comparison with infected macrophages.

Because *L*. *donovani* impairs MHC-II molecules expression [40], which is regulated by IFN-γ [21, 41] and because Ara-LAM induced T cell expansion was dependent on IFN-γRα, we examined whether Ara-LAM would restore MHC-II expression in infected macrophages. Compared to the untreated infected macrophages, Ara-LAM treated infected macrophages had substantially higher MHC-II molecules expression that was further enhanced by rIFN-γ (Fig. 5), corroborating with the Ara-LAM enhanced IFN-γRα-dependent T-cell proliferation in *L*. *donovani*-infected BALB/c mice (Fig. 4F). The Ara-LAM mediated enhanced IFN-γ responsiveness and subsequent improved antigen presentation in parasitized macrophages resulted in abrogated parasitemia (Fig. 5B). Here also Ara-LAM showed greater effectivity in clearing intracellular amastigotes compared to PGN, a known TLR2 ligand, both in vitro and in vivo (S1 and S3 Figs.).

**Fig 6:**
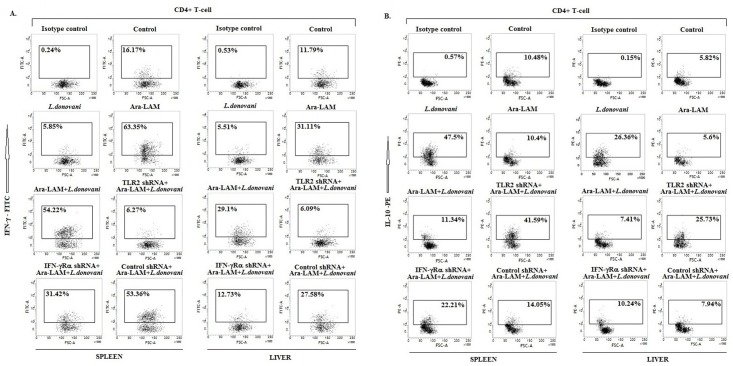
Induction of IFN-γ and IL-10 secreting CD4^+^ T cells by Ara-LAM in *Leishmania*-infected mice. Twenty eight days after infection, different groups of mice were sacrificed, splenocytes and hepatocytes were isolated and stimulated with soluble leishmanial antigen (SLA) for 48hrs. Before harvesting, cells were incubated with brefeldin A (10 mg/mL) for 4 hrs. CD4^+^ T cells were purified by Magnetic Associated Cell Sorter (see Materials and Methods), permeabilized (0.1% saponin) and stained with anti-mouse IFN-γ-FITC (A) and anti-mouse IL-10-PE (B) antibodies and were analyzed by flow cytometry. Data are from one of three experiments conducted in the same way with similar results.

In addition, silencing of TLR2 or IFN-γRα in *L*. *donovani*-infected BALB/c mice reduced the Ara-LAM up-regulated IFN-γ secreting CD4^+^ T cells (Fig. 6A) but increased the Ara-LAM suppressed IL-10 producing CD4^+^ T-cells in both spleen and liver (Fig. 6B).

The data generated from these experiments collectively indicate that Ara-LAM works through TLR2 to restore IFN-γ responsiveness in *L*. *donovani*-infected macrophages and re-establishes host-protective IFN-γ secreting CD4^+^ T cells in a mouse model of visceral leishmaniasis, a fatal disease in absence of an effective therapy.

## Discussion

Visceral leishmaniasis, characterized by severe immunosuppression due to increased parasite-driven Th2 cytokine production [38, 42], is treated with sodium antimony gluconate or miltefosine that have serious side-effects [2, 4]. As these drugs require host immune system for anti-leishmanial activity [32], severe immunosuppression in VL patients reduces their efficacy. As the other anti-leishmanial drugs amphotericin B and paromomycin are not orally bioavailable [43], the treatment becomes more difficult. These problems aggravate with the emergence of drug-resistant *L*. *donovani* [5]. Therefore, finding a suitable immunomodulator is a pressing need for anti-leishmanial therapy.

A class of immunomodulators target TLRs. Several recent findings have suggested the pro-inflammatory role of TLR2 in different infection models [44, 45]. Likewise, Ara-LAM, a potent immunomodulator, uses TLR2 to induce pro-inflammatory functions [10] and to reverse the impaired anti-tubercular cell-mediated immune responses in mouse model [11]. Ara-LAM has been demonstrated as a major component responsible for the pro-inflammatory activity of the whole bacteria (*M*. *smegmatis* and *M*. *fortuitum*) [46]. In an experimental *L*. *donovani* infection, Ara-LAM elicits a strong host-protective pro-inflammatory immune response via TLR2 activated MAPK-mediated signaling, but it fails to do so in TLR2 silenced condition [12, 13]. Although, Ara-LAM is effective in both pre-treatment and post-treatment strategies, the anti-leishmanial effect is more pronounced in pre-treatment model, suggesting Ara-LAM as a immunoprophylactic agent rather than a immunotherapeutic tool against *L*. *donovani* infection [12, 29].

*Leishmania* deactivates the macrophage functions facilitating the parasite infection and propagation within the cell [7, 47]. A key immunomodulatory activity required for eliminating *Leishmania* is IL-12-dependent production of IFN-γ by Th1 cells [14, 15], IFN-γ activates macrophages by iNOS-dependent elimination of the parasite *Leishmania* [16]. Befitting the principle of parasitism, *Leishmania* deactivates macrophages ensuring its intracellular survival [47]. For example, *L*. *donovani* lowers IFN-γ responsiveness in macrophages [7, 48] by lowering IFN-γ receptor expression and by enhancing phosphatase-mediated STAT-1 inactivation [31, 32, 34]. Corroborating to these observations, IFN-γ-deficient mice were more susceptible to *Leishmania* infection than the wild-type controls [49]. These findings implied that targeting TLR for restoring IFN-γ responsiveness could be an immunotherapeutic principle. Therefore, in the present study, we tested whether Ara-LAM, a TLR2 ligand, could restore the IFN-γ responsiveness of the *L*. *donovani*-infected macrophages. We also examined the importance of IFN-γ in Ara-LAM mediated protection against leishmanial pathogenesis. The data presented here indicate that Ara-LAM enhanced transcription of the IFN-γRα gene, perhaps through the recruitment of NF-κB to its promoter (Fig. 1D), and cell surface expression of the receptor in both control and infected macrophages (Fig. 1A-C). Ara-LAM treatment of the *L*. *donovani*-infected cells restored IFN-γ responsiveness, as evident from the Ara-LAM-induced JAK-1, JAK2 and STAT1 phosphorylation in these cells (Fig. 2). As SHP-1 regulates IFN-γRα signaling in infected macrophages [50], the Ara-LAM enhanced IFN-γ responsiveness in *Leishmania*-infected macrophages could also result from reduced SHP-1 phosphorylation. The Ara-LAM induced STAT1 phosphorylation could possibly result from autocrine IFN-γ [12, 32]. Eventually, these Ara-LAM induced events lead to enhanced IFN-γ responsiveness in *L*. *donovani* infected macrophages.

IFN-γ responsiveness also depends on the Interferon regulatory factors (IRFs). Here, we have studied two IRF family members, IRF4 and IRF8, due to their structural semblances, direct involvement in IFN-γ signaling and counteracting roles in the immune system. Here, we observed that their expression was reciprocally regulated by *L*. *donovani* but reversed by Ara-LAM in macrophages (Fig. 3). In *L*. *donovani*-infected macrophages, IRF4 expression and its binding to the IL-10 promoter was induced, whereas IRF8 expression and binding to the IL-12p40 promoter was inhibited. We observed that Ara-LAM increased IL-12 production by effective binding of IRF8 to the IL-12p40 promoter (Fig. 3C-D). The observation is consistent with the reported counteracting roles of IRF4 and IRF8 in the immune system. IRF4 mediates the production of the anti-inflammatory cytokine IL-10 [25]. By contrast, IRF8 mediates the IFN-γ triggered TNF-α, IL-12 and NO production [20] and contributes to the cross-talk between TLR and IFN-γ signaling pathways [28]. Moreover, Ara-LAM enhanced MHC-II expression on the surface of the parasitized macrophages was reinforced by rIFN-γ, possibly through IRF8 (Fig. 5A) [21], for better parasite clearance from the infected macrophages (Fig. 5B). These effects of the IFN-γ activated IRF8 may collectively skew the T cell response to Th1-type, as observed here with Ara-LAM treatment (Figs. 3, 4, 6).

Although complete abrogation of the Ara-LAM mediated host protective immune response and parasite killing in TLR2 silenced mice clearly suggest that Ara-LAM is a TLR2 ligand and it works through TLR2 signaling pathway, for better understanding we compared the effect of Ara-LAM with a known TLR2 ligand, Peptidoglycan (PGN). Here, we observed that both Ara-LAM and PGN induced the IFN-γRα associated JAK1/2-STAT1 signaling cascade in parasitized macrophages resulted in diminished parasitemia, both in vitro and in vivo, but Ara-LAM was found more potent (S1 and S3 Figs.).

Thus, Ara-LAM enhanced IFN-γRα expression, NO generation and Th1 cytokine production along with higher CD4^+^ T-cell activation and proliferation in *Leishmania* infected BALB/c mice. All these host-protective mechanisms were significantly suppressed by IFN-γRα silencing due to absence of effective IFN-γ signaling (Figs. 3, 4, 6). Collectively our findings, both in vitro and in vivo, imply active involvement of IFN-γ signaling in Ara-LAM mediated anti-leishmanial activity.

Thus, we characterize for the first time that Ara-LAM improves the IFN-γ responsiveness of the parasitized macrophages and this IFN-γ signaling, in turn, plays an important role in generating Ara-LAM mediated host protective anti-leishmanial immune response.

## Supporting Information

S1 Fig (A) BALB/c derived peritoneal macrophages (2x10^6^cells) were pre-treated with Ara-LAM (3μg/ml) and PGN (5μg/ml) for 3 hrs and infected with *L*. *donovani* (cell: parasite 1:10) for 24hrs, followed by cell lysate preparation and Western blot for IFN-γRα, JAK1, p-JAK1, JAK2, p-JAK2, STAT-1, p-STAT-1 and GAPDH. The blots shown are representative of triplicate experiments. (B) Both the nuclear extracts and the cytosolic extracts of differently treated peritoneal macrophages were prepared followed by Western blot to analyze the nuclear translocation of p-STAT-1 in *L*. *donovani* infected macrophages. Blots shown here are from one of three representative experiments. (C) In a separate experiment, the macrophages were cultured in cover slips, treated with Ara-LAM and PGN for 3 hrs followed by *Leishmania* infection. After 24hrs of incubation intracellular parasite number were assessed as described in methods. Data represent means ± SD for three sets of experiments. ***P <.001 for the comparison with infected macrophages.
(TIF)

S2 Fig Mice were injected with TLR2 or IFN-γRα shRNAs (for 2 days) followed by treatment with phosphate-buffered saline (PBS) (control). After 30 days mice were sacrificed, splenocytes (2x10^6^) was collected in Trizol and cell lysis buffer for mRNA and protein extraction respectively. TLR2 and IFN-γRα expressions were studied by semi-quantitative RT-PCR and Western blot methods (see Methods). Each data are from one of three representative experiments.
(TIF)

S3 Fig Mice were treated with either Ara-LAM (30 μg intraperitoneally) or PGN (50 μg intraperitoneally) for 2 days, followed by *L*. *donovani* infection. After 28 days mice were sacrificed. Levels of parasite burden in liver and spleen are expressed in Leishman-Donovan units (LDUs). Data represent means ± SD for 4 animals per group. ***P <.001 for the comparison with infected mice.
(TIF)
