# Expression of Concern: The host-protective effect of arabinosylated lipoarabinomannan against *Leishmania donovani* infection is associated with restoration of IFN-γ responsiveness

**DOI:** 10.1371/journal.pone.0350442

**Published:** 2026-06-02

**Authors:** The PLOS One Editors

After this article [1] was published, concerns were raised regarding results presented in Fig 1 and the description of the animal experiments.

Specifically:

- Fig 1B GAPDH lanes 1-5 in [1] appear similar to the Fig 3B GAPDH panel in [2,3] despite being used to represent different experimental conditions.
- The Materials and Methods section does not report the method of euthanasia used in the animal experiments.
- Contrary to the article’s Data Availability statement, the original raw data files supporting the article’s results were not provided with the article.

The first author disagreed with the journal’s observations that results in the Fig 1B panel in [1] appear similar to results in Fig 3B in [2,3] and stated that the original image data for this figure are no longer available. In the absence of these data, this concern cannot be resolved and the reliability of the associated quantified results cannot be verified.

Regarding the method of euthanasia, the first author stated that during the experiments conducted at the Bose Institute, mice were euthanized using standard and approved methods, including CO₂ inhalation, with cervical dislocation employed as a secondary method when required. They stated a portion of the animal work was carried out at the National Centre for Cell Science (NCCS), Pune and that all animal experiments conducted at both the Bose Institute and NCCS were performed in accordance with the guidelines approved by the respective Institutional Animal Ethical Committees. The first author stated that the IACUC ethical approval number from NCCS is no longer available.

The first author stated the original underlying data cannot be provided as the laboratory has closed since the publication of the article [1], and the original files are no longer accessible. As the original raw data files supporting the article’s results have not been made available, this article [1] does not comply with the PLOS Data Availability policy in place at the time this article was submitted**.**

The *PLOS One* Editors issue this Expression of Concern to inform readers of the above unresolved concerns and that the results presented in Fig 1B should be interpreted with caution.
