# Supplementary figures and images for: The Host-Protective Effect of Arabinosylated Lipoarabinomannan against Leishmania donovani Infection Is Associated with Restoration of IFN-γ Responsiveness
Source: PLoS One. 2015 Feb 6;10(2):e0117247. doi: 10.1371/journal.pone.0117247 (PMC4319725; doi:10.1371/journal.pone.0117247)

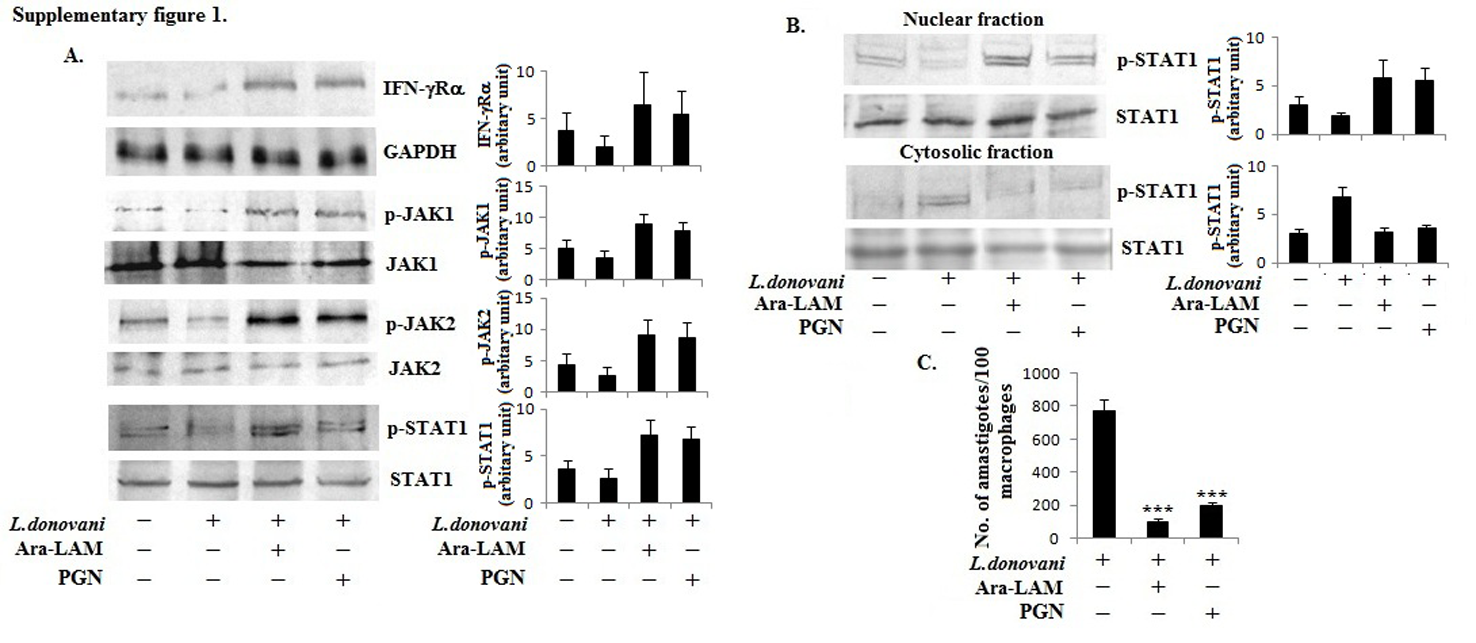

Supplement: S1 Fig — The blots shown are representative of triplicate experiments. (B) Both the nuclear extracts and the cytosolic extracts of differently treated peritoneal macrophages were prepared followed by Western blot to analyze the nuclear translocation of p-STAT-1 in L. donovani infected macrophages. Blots shown here are from one of three representative experiments. (C) In a separate experiment, the macrophages were cultured in cover slips, treated with Ara-LAM and PGN for 3 hrs followed by Leishmania infection. After 24hrs of incubation intracellular parasite number were assessed as described in methods. Data represent means ± SD for three sets of experiments. ***P <.001 for the comparison with infected macrophages. (TIF) [file pone.0117247.s001.tif]

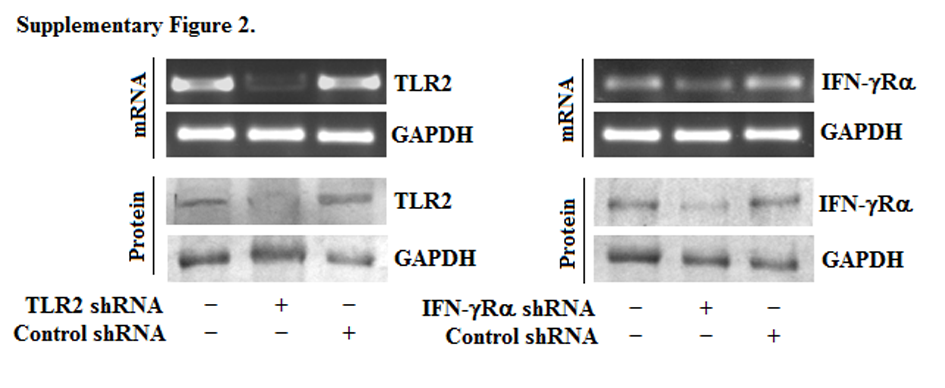

Supplement: S2 Fig — After 30 days mice were sacrificed, splenocytes (2x10^6^) was collected in Trizol and cell lysis buffer for mRNA and protein extraction respectively. TLR2 and IFN-γRα expressions were studied by semi-quantitative RT-PCR and Western blot methods (see Methods). Each data are from one of three representative experiments. (TIF) [file pone.0117247.s002.tif]

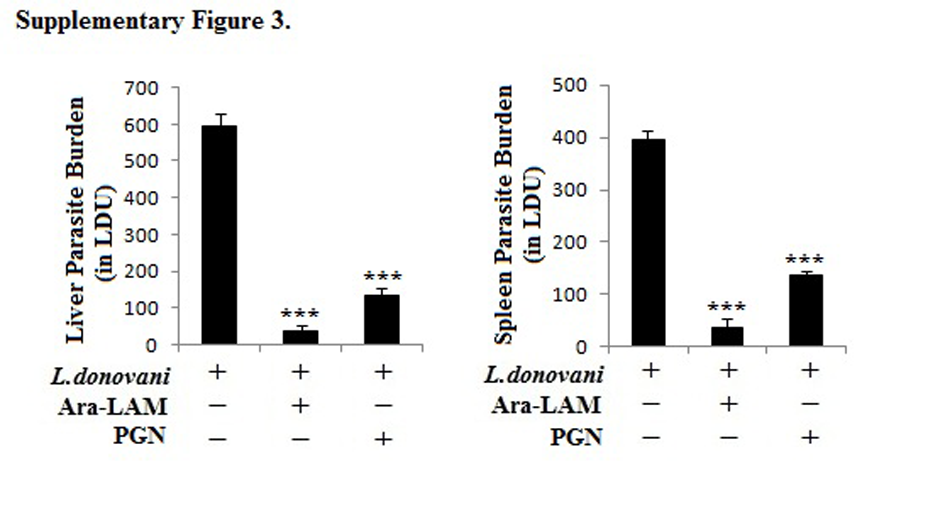

Supplement: S3 Fig — After 28 days mice were sacrificed. Levels of parasite burden in liver and spleen are expressed in Leishman-Donovan units (LDUs). Data represent means ± SD for 4 animals per group. ***P <.001 for the comparison with infected mice. (TIF) [file pone.0117247.s003.tif]
